# Polycystic ovary syndrome resembling histopathological alterations in ovaries from prenatal androgenized female rats

**DOI:** 10.1186/1757-2215-5-15

**Published:** 2012-05-18

**Authors:** Fang Wang, Bolan Yu, Wenjing Yang, Jianqiao Liu, Jiachun Lu, Xuefeng Xia

**Affiliations:** 1 Institute of Gynecology and Obstetrics, the Third Affiliated Hospital of Guangzhou Medical University, Guangzhou, 510150, China; 2 Wuhan 6th People’s Hospital, Wuhan, 430015, China

**Keywords:** Prenatal androgenization, Polycystic ovary syndrome (PCOS), Ovarian, Rat model

## Abstract

**Background:**

The polycystic ovary syndrome **(**PCOS**)** affects approximately 6-10% of women of reproductive age and is characterized by chronic anovulation and hyperandrogenism. However, a comprehensive understanding of the mechanisms that dictate androgen overproduction is lacking, which may account for inconsistencies between measures of androgen excess and clinical presentation in individual cases.

**Methods:**

A rat model of PCOS was established by injecting dehydroepiandrosterone sulfoconjugate (DHEAS) into pregnant females. Rats were administered with DHEAS (60 mg/kg/d) subcutaneously (s.c.) for all 20 days of pregnancy (Group A), or for the first 10 days (Group B), or from day 11 to day 20 (Group C). Controls were administered with injection oil (0.2 ml/day) s.c. throughout pregnancy (Group D). The litter rate, abortion rate, and offspring survival rate in each group were recorded. Serum androgen and estrogen were measured and the morphological features of the ovaries were examined by light and electron microscopy in the offspring of each group.

**Results:**

We found that rats injected with DHEAS throughout pregnancy (group A) lost fertility. Rats injected with DHEAS during early pregnancy (group B) exhibited more serious aberrations in fertility than both Group C, in which rats were injected with DHEAS during late pregnancy (P < 0.05), and Group D (controls). There was a statistical difference of ovarian weight among female offspring in Group B, C and D (P < 0.01). By light and electron microscopy, a significant morphological difference among the female offspring in the three groups was observed.

**Conclusions:**

Our results indicate that androgen excess during pregnancy can decrease rat fertility. Excess androgen at the early stage of pregnancy causes high reproductive toxicity, leading to abnormality of ovarian morphology and functions in female offspring.

## Background

Polycystic ovary syndrome (PCOS) is the most common endocrine disorder in premenopausal women [1]. In different ethic populations, the prevalence of PCOS is about 5-10% [2,3]. In clinic, PCOS patients are usually diagnosed by a distinguishing polycystic appearance of the ovary and a wide range of biochemical markers including elevated serum androgens, insulin, luteinizing hormone (LH), and decreased insulin sensitivity [4]. Both healthy women and women with abnormal cycles and hyperandroenism could develop PCOS, however, overweight or obese girls are much more predisposed to PCOS [4,5].

Currently, the etiology of PCOS remains largely unknown. It is believed that both genetic factors and gestational environment contribute to its origin and development, and prenatal androgen exposure is considered one of the important factors in the early origins of PCOS [1,6,7]. It has been found that adrenal androgen excess presents in approximately 25-60% of women with PCOS, in which dehydroepiandrosterone (DHEA), dehydroepiandrosterone sulfoconjugate (DHEAS), and androstenedione are the most common androgens that are elevated [8,9]. Epidemiological studies reported that girls at high risk for PCOS may have early androgen excess [10,11]. Experiments with nonhuman primate model for PCOS demonstrated that prenatally androgenized female monkeys had enhanced basal and adrenocorticotropic hormone (ACTH)-stimulated adrenal androgen levels [8,12-15]. In sheep, exposure of the pregnant ewe to large doses of testosterone causes increased LH secretion and abnormal ovarian cycles in female offspring [16-18]. Recently, studies proved that *in uetro* androgen exposure in adult female rats could also develop the PCOS related metabolic syndrome [19,20]. Therefore, a strong association of PCOS development and prenatal androgen excess were well supported by literature.

The mechanisms for PCOS development caused by the prenatal androgenization are still under investigation. Human studies suggest continuing actions of hyperandrogenemia are important for sustaining the abnormal hypothalamic sensitivity to feedback inhibition by ovarian steroids [21]. In cultured human theca cells from PCOS patients, 20 times more androstenedione was produced than normal cells [22,23]. In rat models, prenatal androgenization leads to higher progesterone receptor mRNA expression and more LH secretion, prolonged anogenital distance and fewer nipple numbers, irregular or prolonged estrous cycles [20]. Therefore, a global change in the reproductive system and ovarian functions in PCOS patients is suggested. As animal models are useful in understanding the pathogenesis of PCOS, it is important to examine the histopathological alterations in ovaries from prenatal androgenized animal models [24].

In current study, we established a rat model of PCOS by injecting androgen into pregnant females. The weight, morphology, and functions of the ovaries of female offspring in different treated groups were compared, and the detailed histopathological alterations in ovaries of prenatal androgenized female rats were examined. The major goal of this study is to determine the impact of prenatal androgenization on ovary development in female offspring, which is lacked in most reported literatures, and to provide new insights into the mechanisms of prenatal androgenization and PCOS development.

## Methods

### Animals and drugs

The study protocol was approved by the institutional Animal Care and Use Committee of Guangzhou Medical University. Rats were purchased from and housed in the Animal Center of Guangzhou Medical University. DHEAS and injection oil were purchased from Hubei Fangtong Pharmaceutical Co., Ltd. The rats were maintained on a controlled light cycle of 14:10 h (light/dark) at 25°C with water and food *ad libitum*. All animal procedures were reviewed and approved by the Guangzhou Medical University before implementation.

### Administration of DHEAS

Rats were administered with DHEAS between 8:30 and 10:30 a.m. DHEAS (60 mg/kg/d) was dissolved in 0.2 ml injection oil and injected subcutaneously (s.c.) in the inner thighs of rats. The first day of pregnancy was defined by first sight of a vaginal plug. Thirty-two female SD rats (16-week-old, weighing 180–200 g) were randomly subdivided into 4 groups (Group A-D) for different treatments. Group A: rats were injected with DHEAS (60 mg/kg/d) during the whole pregnancy (day 1 to day 20). Group B: rats were injected with DHEAS (60 mg/kg/d) during the first half pregnancy (day 1 to day 10). Group C: rats were injected with DHEAS (60 mg/kg/d) during the second half pregnancy (day 11 to day 20). Group D (control): rats were injected with oil only (day 1 to day 20). The litter rate, abortion rate, and offspring survival rate were recorded. If abortion occurred, the rat was impregnated again after 1–2 weeks. The female offspring of each group were Group A’, B’, C’, and D’ accordingly, and their serum androgen and estrogen were measured and the morphological features of the ovaries were examined by light and electron microscopy. Experiments were repeated for at least 3 times, average results or representative figures were analyzed.

### Determination of estrus cycle, pregnancy, and rate abortion

The estrus cycle was determined by vaginal exfoliative cytology and the ovulation was observed. The estrus cycle of rats is approximately 4–5 days, including proestrus, estrus, metestrus, and anestrus. The pregnancy period of rats is 21 days on average from first sight of the vaginal plus, and ranges from 19–23 days. To measure vaginal exfoliation, a cotton swab soaked in saline was inserted into the vagina, rotated, and the adherent mucous tissue smeared on slides for hematoxylin and eosin (HE) staining. The morphologic features of vaginal exfoliative cells were examined by light microscopy.

Early abortion, in which prenatal rats were not formed, was judged by weighing the dames, and by examining maternal bodies to see if abdomens were enlarged and if breasts were appropriately developed. No increase in body weight or reduced body weight, absence of abdominal enlargement, and lack of breast development were taken as signs of early abortion. Late abortion was measured by dead bodies in the cage. If the external genitalia of the maternal body or cushions were stained with blood, the offspring may have been eaten, which was also considered to be abortion. Offspring survival rates were obtained by dividing the number of adolescent rats by the number at birth in each group.

### Measurement of serum sex hormones

The serum levels of sex hormone were determined by microparticle chemiluminescent immunoassay kits (Beckman Corporation of America) according to the manufacturer’s instructions. Intraday and interday coefficients of variation were below 10%. Three independent experiments were performed and the results were compared by independent sample *t*-test using SPSS 13.0 (SPSS Inc. USA). A two-sided *P* value of 0.05 was considered statistically significant.

### Tissue sample collection and histochemistry

The offspring were sacrificed on the seventh day of the 10^th^ week (equivalent to adolescence), and serum samples and ovaries were collected. Blood samples were collected by cardiac puncture, centrifuged at 2000 rpm, and plasma was collected and stored at −20°C for hormone analysis. After taking blood samples, both ovaries were quickly removed, cleared up and weighed. The visible morphologic changes of both ovaries including color and texture were recorded. After that, one ovary was quickly rinsed in phosphate buffered saline (PBS), fixed in 2.5% glutaraldehyde solution, and stored at 4°C for further processing and transmission electron microscopy, while the other ovary was fixed in 10% formalin solution and stored at 4°C for HE staining and light microscopic examination. Partly wax-enveloped ovarian tissues were stained with HE and the growth of follicles observed by microscopy and compared between offspring groups. The contralateral ovaries were prepared for transmission electron microscopy, and changes in their cellular and subcellular structures were analyzed and compared.

## Results

### Litter rate, abortion rate, and offspring survival rate

During the first round of androgen treatment, pregnant rats in Groups A, B, and C had abortion rates of 100%, while the rate is 0% in Group D (controls). After abortion, the rats were kept in the same cage. No pregnancy occurred in Group A after 10 estrus cycles. Exfoliative cytoscopy of vagina showed that typical periodic changes in Group A rats were absent, while Groups B and C were able to become pregnant and carry litters to term. The offspring survival rate was 65% in Group B, 70.97% in Group C, and 71.43% in Group D. There was a statistical difference in survival rate between Group B and C (P < 0.05), and between Group B and D (P < 0.05) by independent sample *t*-test. No statistical difference in survival rate was found between Group C and D (P > 0.05).

### Anatomic changes in offspring ovaries

The ovaries of Group B’ and C’ were slightly larger than those of Group D’. Some of ovaries of Group B’ and C’ were slightly red on the surface and no obvious vesicae were observed. The others were pale on the surface, and many vesicae were observed, especially in Group C’. The ovaries of Group D’ were uniformly red in color. The ovarian weight was 0.029 ± 0.007 g in Group B’, 0.030 ± 0.006 g in Group C’, and 0.026 ± 0.007 g in Group D’. There was a statistical difference between Group D’ and the combined Group B’ and C’ (P < 0.05), but no significant difference between Groups B’ and C’(P > 0.05).

### Observation of ovaries in neonatal rats by light microscopy

**Figure 1:**
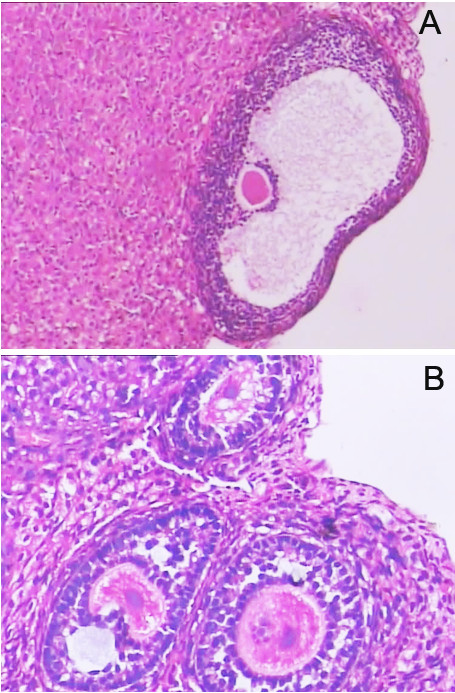
**Histological examination of normal ovaries.A** and **B.** light microscopy photographs of ovaries from rat offspring in the controls (HE × 200).

**Figure 2:**
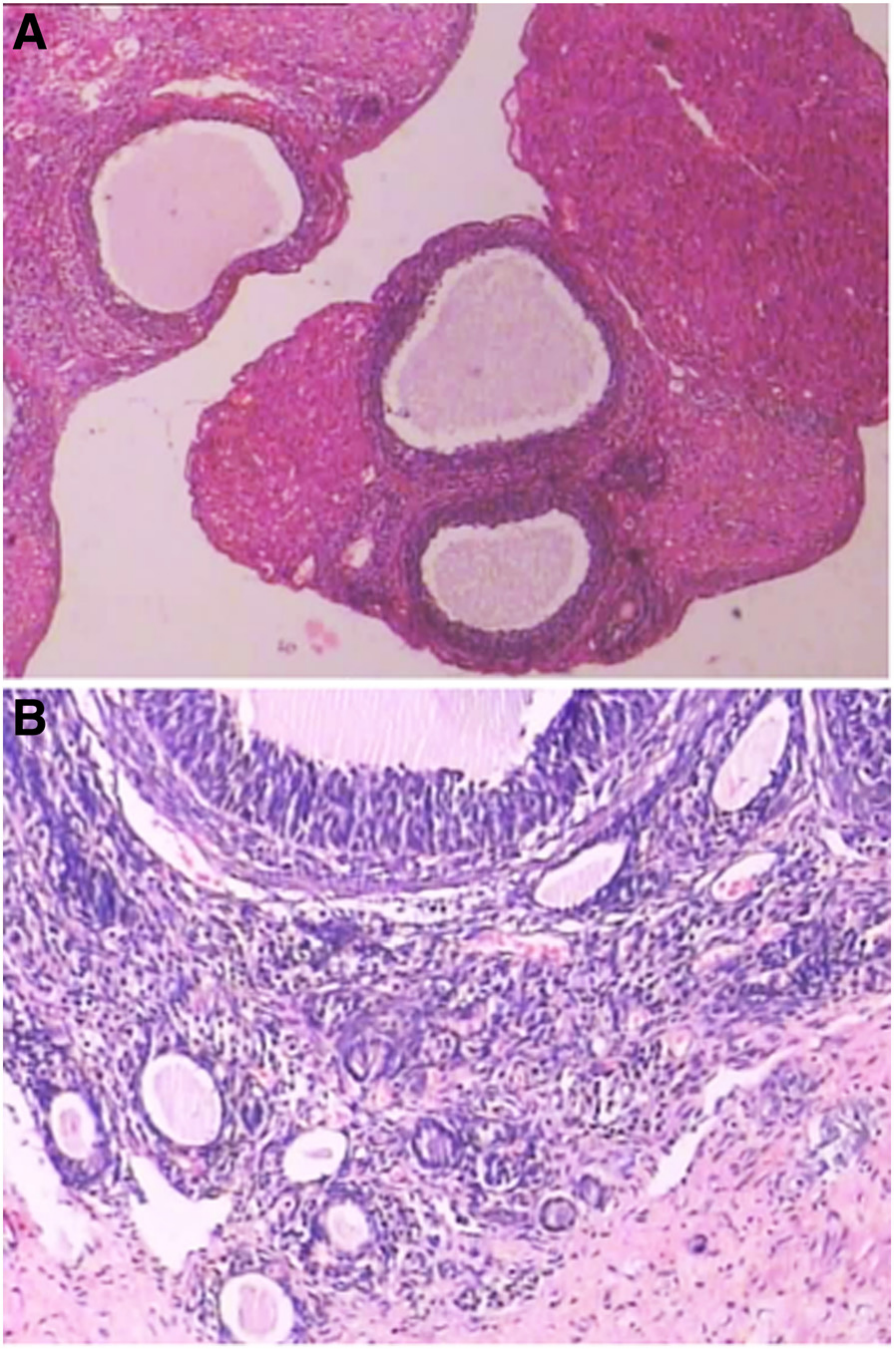
**Representative histopathological alterations in ovaries of prenatal androgenized female rats.A.** PCOS resembling changes with decreased granular cells in ovaries of rat offspring in the Group B and C (HE × 100). **B.** Preantral follicles and cystic follicles in ovaries of rat offspring in the Group B and C (HE × 200).

The corpora lutea and follicles at different developmental stages were examined in ovaries by light microscopy. Multiple layers of granular cells were observed in offspring in Group D’ (Figure 1), while the number of granular cell layers was significantly lower in offspring in Group B’ and C’ (Figure 2A). In contrast, the number of theca cell layers was greater than in offspring in Groups B’ and C (Figure 2). In Groups B’ and C’, primary follicles and primordial follicles increased in diameter and contained numerous cystic preantral follicles and cystic follicles (Figure 2B). Oocytes and corona radiata were absent and the follicles were surrounded by hyperplastic luteinized cells of the follicular inner membrane. Hyperplasia of mesenchymal cells in the ovary cortex was also obvious (Figure 2).

### Observation of ovaries in neonatal rats by electron microscopy

**Figure 3:**
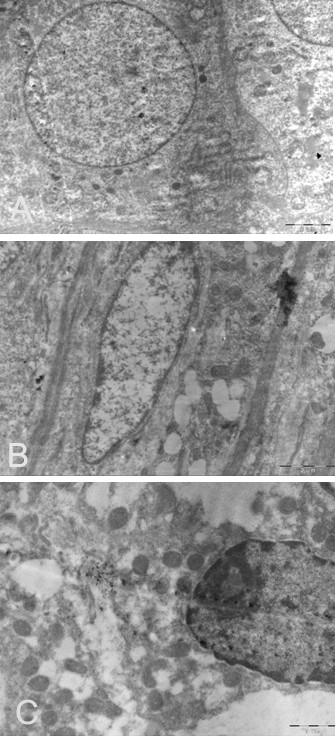
**Electron micrographs of ovaries from rat offspring in different groups.A.** ovaries from rat offspring in the controls (TME × 12000). **B.** ovaries from rat offspring in the Group B and C. Arrows indicated the granular cells (TME × 12000). **C.** ovarian granular cells of rat offspring in the Group B and C (TME × 12000).

Mitochondria, microtubules, rough and smooth endoplasmic reticulum, and some lipid drops were observed in the control group by electron microscopy (Figure 3). Organelles involved in the synthesis of proteins were abundant in the granular cells and follicular cells of Groups B’ and C’; the number of mitochondria was higher than controls but the mitochondrial crista were blurred and faded (Figure 3B). Some mitochondria were spherical and pyknotic, swollen or even ruptured, replaced by lipid drops and vesicae. Ribosomes were abundant but smooth endoplasmic reticulum was not. Enlarged nucleoli, irregular nucleoli, reduced nucleoplasm, shrunken nuclear membranes, and local swelling were observed (Figure 3B). Some oocytes had uneven cytoplasm in which mitochondria were swollen and appeared fused to lysosomes. The rough endoplasmic reticulum was expanded, degranulated, disorganized, and even showed signs of rupture. Golgi bodies were also swollen and their boundaries blurred. Few polyribosomes were observed, local cytoplasmic lamellar structure was absent (Figure 3B). Numerous lipid droplets and vacuoles were observed in granular cells and in the cytoplasm of cells of the follicular membrane (Figure 3C).

### Serum androgen and estrogen levels

The serum concentrations of follicle stimulating hormone (FSH) and LH were below 0.5 IU/L, and prolactin (PRL) were below 0.5 ng/mL in Groups B', C', and D' (data not shown). The serum testosterone level was significantly higher in the treated groups (B' and C') than in the control group (P < 0.01). In contrast to serum testosterone, serum estradiol (E_2_) was lower in Group C' than Group B' (P < 0.01). Group B' had the highest levels of serum estradiol, and group C’ had the highest levels of serum testosterone in the three groups (Table 1).

**Table 1:** **Comparison of serum hormone levels among Group B’, C’, and D’**^a^

Group	N	Testosterone (ng/ml)	Estradiol (pg/ml)
Group B'	35	3.84 ± 0.63^b^	50.11 ± 7.98^d^
Group C'	39	9.07 ± 1.42^c^	30.67 ± 6.89^ef^
Group D'	42	2.59 ± 0.42	39.56 ± 3.75

^a^: Group B’: female offspring of rats injected with DHEAS (60 mg/kg/d) during the first half pregnancy. Group C’: female offspring of rats injected with DHEAS (60 mg/kg/d) during the second half pregnancy. Group D’ (control): female offspring of rats injected with oil only.

^b^: P < 0.01 compared to Group D'.

^c^: P < 0.01 compared to Group B' or Group D'.

^d^: P < 0.01 compared to Group D'.

^e^: P < 0.05 compared to Group D'.

^f^: P < 0.01 compared to Group B'.

## Discussion

In this study, we demonstrated that elevated androgen levels during pregnancy significantly disrupted pregnancy and leaded to a PCOS-like syndrome in the female offspring. No pregnancies occurred in rats receiving daily injections of androgens throughout pregnancy, even after 10 estrus cycles. Exfoliative cytoscopy of their vagina showed no typical periodic changes associated with estrus, while rats treated with androgens during the first and second halves of pregnancy were able to undergo pregnancy after an initial period of infertility. Therefore, consisting with earlier reports [19,20,25], our data showed that high androgen levels during pregnancy had significant negative impacts on fertilization and could lead to permanent histopathological alterations in ovaries of female offspring (Table1, Figure 1,2,3).

Our results demonstrated that the offspring survival rate of rats treated with androgens during the first halves of pregnancy was lower than that of rats treated with androgens during the second halves of pregnancy or controls (P < 0.05). Our data was consist with studies of Dumesic *et al.*, who reported that rhesus monkeys exposed to high androgen during the first trimester showed deficient oocyte development compared to mothers treated during the third trimester [15]. It has been found that oocytes collected from rhesus monkeys with early prenatal androgenization were normally fertilized but abnormal development occurred after 8 cell phases compared to the control group [26], partially due to prematurity and aging of oocytes at ovulation and corresponding high spontaneous abortion rate. The reason for this might come from hormone disruption by androgen in the early stage of embryo development. It was known that high androgen levels during the first trimester disrupted fetal gonad differentiation, pancreas formation, and neuroendocrine development. During the first half of pregnancy, androgen administration increased the secretion of Gonadotropin-releasing hormone (GnRH) in the fetuses, which might also elevate maternal GnRH through the umbilical arteriovenous circulation and disrupt the whole maternal hypothalamus-pituitary-gonadal axis. Therefore, mothers exposed to high androgen during the first trimester might suffer more adverse effects on fertility than those exposed during the second and third trimesters.

In this experiment, there were significant differences in ovary weights and morphology between controls and rats treated with androgens during the first and second halves of pregnancy. Significant morphologic differences between the androgen-treated groups and the control group were observed by light microscopy while changes in organelle structures were observed by electron microscopy (Figure 1, 2, 3). In addition, excessive theca cells caused an elevation in serum sex hormones (Table 1). Since organ morphology is always linked with its function, the PCOS resembling histopathological alterations in ovaries of prenatal androgenized female rats suggested that androgen affects the ovary development first and consequent functions later. Because the aggregation of follicles and the morphologic changes are important characteristic in PCOS develop, our observation supported that these morphologic changes might result from androgen excess.

High androgen during early pregnancy, similar to congenital adrenal hyperplasia, is characterized by increased secretion of LH, which causes secondary ovarian hyperandrogenism. It is believed that prenatal exposure to high androgen activates the expression of androgen receptor genes in the preoptic area of the fetal hypothalamus and inhibits the estrogen-induced expression of progesterone receptor genes or even causes permanent impairment in receptor expression. Thus, secretion pattern of GnRH is no longer feminine; rather, the basic GnRH pulse generator is characterized by frequent secretion but fails at a secretion higher frequency when the E_2_ peaks. Previous studies have observed metabolic disturbances in adult offspring. Newborn rats prenatally exposed to high androgen during the third trimester developed hyperandrogenism but no obvious metabolic syndrome as evidenced by normal body weights and the oral glucose tolerance test (OGTT). The serum androgen concentration in Group C' was significantly higher than the other groups (Table 1). In addition, organelles involved in the synthesis of proteins (rough ER and Golgi) significantly increased but their morphologic structures were impaired (Figure 3), which might be related to the imbalance in hormone synthesis.

Abnormal metabolism in women may be unrelated to hyperandrogenism following clinical development of PCOS; rather, it reflects an ovarian structural abnormality that causes dysfunction of the hypothalamus-pituitary-growth axis or pancreatic islands, leading to both abnormal metabolism and hyperandrogenism. Since functions are based on structures, and ovarian structural abnormality leads to dysfunction, further research is required to determine the details in PCOS development and to improve the symptomatic treatment for this disease.

## Conclusions

In this study, our data supported that androgen excess severely impact female rat fertility. Using a rat model of PCOS by injecting DHEAS into pregnant females during different stages, it was found that rats injected with DHEAS throughout pregnancy completely lost fertility, and rats treated during early pregnancy exhibited more serious aberrations in fertility than rats treated during late pregnancy compared to controls. Besides elevated androgen levels during pregnancy, there were significant increases of ovary weights in offspring of rats treated with DHEAS. The female offspring of androgen-treated rats also exhibited significant morphologic differences and organelle structure changes in ovaries. These PCOS resembling histopathological alterations in ovaries of prenatal androgenized female rats suggested that androgen affects both ovary development and its function. Therefore, our study indicated excess androgen during pregnancy, especially at the early stage, may cause high reproductive toxicity and abnormal morphology and functions of ovaries in female offspring.

## Abbreviations

PCOS: polycystic ovary syndrome; DHEAS: dehydroepiandrosterone sulfoconjugate; DHEA: dehydroepiandrosterone; ACTH: adrenocorticotropic hormone; LH: luteinizing hormone; PBS: phosphate buffered saline; FSH: follicle stimulating hormone; PRL: prolactin; GnRH: Gonadotropin-releasing hormone; E2: estradiol; OGTT: oral glucose tolerance test.

## Misc

Fang Wang and Bolan Yu contributed equally to this work

## Competing interests

The authors declare that they have no competing interests.

## Authors’ contributions

FW, BY and XX designed the study. FW and BY wrote the manuscript. FW and WY performed experiments and analyzed data. JL and JL made critical revision of the manuscript. All authors read and approved the final manuscript.
